# Sport Motivation and Mental Health Outcomes Among Padel Players in Saudi Arabia: A Cross-Sectional PLS-SEM Study

**DOI:** 10.3390/sports14070280

**Published:** 2026-07-03

**Authors:** Yousef Saad Aldabayan, Ibrahim A. Elshaer, Youssef Kooli, Mansour Alyahya, Chokri Kooli

**Affiliations:** 1 Department of Management, School of Business, King Faisal University, Al-Ahsa 31982, Saudi Arabia; 2 Department of Respiratory Care, College of Applied Medical Sciences, King Faisal University, Al-Ahsa 31982, Saudi Arabia; 3 Department of Human Kinetics, College of Health Sciences, University of Ottawa, Ottawa, ON K1N 6N5, Canada; 4 Department of Management, Faculty of Social Sciences and Humanities, Royal Military College of Canada, Kingston, ON K7K 7B4, Canada; 5 Graduate School of Public and International Affairs, Faculty of Social Sciences, University of Ottawa, Ottawa, ON K1N 6N5, Canada

**Keywords:** Padel sport, intrinsic motivation, extrinsic motivation, stress, depression, anxiety, mental health

## Abstract

The rapid evolution of Padel in Saudi Arabia (SA) has positioned the sport as a popular recreational and social activity, mainly among young adults. However, limited research has examined how different forms of sport motivation are associated with mental health outcomes in this emerging context. Drawing on Self-Determination Theory (SDT), this study investigated the associations between intrinsic and extrinsic motivation and depression, stress, and anxiety among Padel players in SA. A quantitative, cross-sectional online survey was conducted with a sample of 475 players, the majority of whom were aged 17–35 and held at least a bachelor’s degree. Data were analysed using Partial Least Squares Structural Equation Modelling (PLS-SEM) to evaluate the relationships between multidimensional motivation factors and mental health symptoms. The findings revealed a nuanced, at times paradoxical, pattern of relationships. Intrinsic motivation to experience stimulation (engaging in an activity because of the positive sensations, excitement, enjoyment, or stimulation that the activity itself provides, rather than for external rewards or pressures) was consistently associated with lower levels of depression, stress, and anxiety, suggesting that enjoyment-driven involvement is associated with better mental health outcomes. In contrast, intrinsic motivation to accomplish was positively correlated with all three mental health indicators, indicating that achievement-oriented engagement might intensify emotional pressure. Among extrinsic motivations, external regulation was significantly associated with poorer mental health outcomes. In contrast, introjected regulation unexpectedly displayed a negative association with psychological distress, demonstrating a potentially adaptive role in this setting. Identified regulation, however, was not significantly associated with any mental health symptoms. These results underscore the “double-edged” nature of sport motivation, showing that not all internal or external motives yield uniformly positive consequences. The study contributed to the growing literature by providing a context-specific understanding of how motivational dynamics function within a rapidly growing sport in Saudi Arabia. In practice, the findings suggested that enjoyment-based involvement was associated with more favourable mental health outcomes, whereas performance-related pressures might be associated with less favourable outcomes.

## 1. Introduction

Frequent engagement in physical activity, exercise, and sports has consistently been associated with better emotional well-being and more favourable mental health outcomes across different age groups [1,2,3,4,5,6,7]. For example, an extensive body of literature has revealed that engagement in sports activities can contribute to reduced symptoms of stress, anxiety, and depression, while concurrently improving psychological regulation, perceived self-esteem, and overall well-being [3,8]. Recently, a lot of attention has shifted from simply investigating the intensity or frequency of physical engagement to exploring the motivational drivers that energise ongoing participation in sports and their differential associations with mental health consequences [9,10,11,12]. Sports motivation can be conceptualised within the context of Self-Determination Theory (SDT), which distinguishes between intrinsic and extrinsic motivation based on the level of self-determination [13]. Self-Determination Theory (SDT) was selected as the theoretical foundation of this study because it offers one of the most comprehensive frameworks for understanding why people initiate and sustain participation in sport activities and how different motivational regulations can be associated with psychological outcomes [14,15]. Unlike other motivational frameworks, such as Achievement Goal Theory, which primarily focuses on mastery and performance orientations, or the Theory of Planned Behaviour, which emphasises behavioural intentions and attitudes, SDT distinguishes between multiple forms of intrinsic and extrinsic motivation based on their degree of self-determination [16]. This distinction is particularly relevant for understanding mental health outcomes, as previous research has shown that autonomous forms of motivation are generally associated with greater psychological well-being. In contrast, controlled forms of motivation are often linked to psychological distress. Moreover, SDT has been extensively validated within sport and exercise psychology and serves as the theoretical basis for the Sport Motivation Scale (SMS), the instrument employed in this study [17]. Therefore, SDT provides a theoretically coherent and empirically robust framework for examining how different motivational dimensions are associated with depression, anxiety, and stress among Padel players in Saudi Arabia.

Within this context, intrinsic motivation is driven by internal enjoyment, mastery, or stimulation and has been consistently correlated with a higher level of psychological well-being, whereas extrinsic motivation is driven by external rewards, challenges, or pressures [18]. Previous empirical evidence argued that professional and recreational sport participants who are motivated intrinsically showed lower levels of stress, depression and anxiety, higher levels of psychological resilience, and were associated with better levels of stress control compared to those driven mainly by external obligations or rewards [19,20,21]. Despite this well-established evidence, much of the current literature has focused mainly on traditional sports and physical activities, with limited attention paid to emerging racket sports, such as Padel, which have seen rapid global participation growth over the past few years. Padel is recognised as a fast-growing sport that combines the rules of tennis and squash and is characterised by high levels of social interaction, moderate-to-vigorous physical activity (MVPA), and greater recreational appeal [22,23,24]. The growing popularity of Padel among young athletes and adults makes it a potentially valuable setting for supporting mental health through sport engagement [25]. However, empirical studies testing the motivational and psychological aspects of Padel remain limited. Specifically, limited knowledge exists regarding how different types of sport motivation among Padel players may be related to mental health outcomes.

While intrinsic motivation has frequently been associated with positive psychological outcomes, Self-Determination Theory (SDT) also highlights the importance of extrinsic motivation and its various forms of behavioural regulation. Extrinsic motivation is not a uniform construct; rather, it ranges from relatively autonomous forms, such as identified regulation, to more controlled forms, such as introjected and external regulation. Previous research suggests that the psychological consequences of extrinsic motivation depend largely on the degree of internalisation underlying a behaviour [21,26]. More autonomous forms of extrinsic motivation are generally correlated with greater persistence, well-being, and adaptive psychological functioning, whereas highly controlled forms of motivation, particularly those driven by external rewards, social pressure, or avoidance of guilt, have been linked to elevated levels of stress, anxiety, burnout, and psychological distress [27]. Therefore, testing the multidimensional nature of extrinsic motivation is essential for understanding how different motivational regulations may differentially be associated with mental health outcomes among sport participants.

Mental health consequences in sport psychology studies are commonly measured using multidimensional scales that capture people’s psychological states [7]. One of the most broadly employed and psychometrically scale for evaluating mental health disorders is the “Depression Anxiety Stress Scales” (DASS), which measures the main elements of psychological distress, comprising depression, anxiety, and stress (Lovibond & Lovibond [28]). Previous research using the DASS has confirmed that consistent physical activity is associated with lower levels of psychological distress, but the strength and direction of these correlations vary depending on people’s motivations for sport engagement [29,30,31]. Although the association between sport participation and mental health has been extensively examined in the literature, important gaps remain in understanding how different motivational dimensions can be associated with mental health outcomes across specific sporting contexts. In particular, limited empirical evidence exists regarding the relationships between intrinsic and extrinsic forms of motivation and mental health outcomes among Padel players, especially within the Saudi Arabian context. Although motivational theories such as Self-Determination Theory and Achievement Goal Theory have been widely applied in sport psychology research, important gaps remain regarding how different motivational regulations are associated with mental health outcomes across specific sporting contexts. Addressing this issue is important because understanding the motivational factors linked to depression, anxiety, and stress may help sport organisations, coaches, and policymakers develop strategies that promote both sustained sport participation and psychological well-being. Accordingly, the present study extends existing motivational research by examining how distinct motivational dimensions are associated with depression, anxiety, and stress among Padel participants.

Addressing the previous gaps, the current research aims to explore the relationships between sports motivation among Padel players and mental health outcomes, employing the Sport Motivation Scale (SMS; Pelletier et al., [26]) with its six motivational factors and the DASS to measure mental health disorders. By focusing on Padel players, this research sought to extend the sport psychology literature to an underexplored sporting population and to offer empirical insights into the associations between motivational factors and mental health outcomes. From a practical perspective, understanding the motivational processes underlying emotional well-being can help coaches, sport organisations, and health professionals develop motivation-based mechanisms that might be associated with sustained sport involvement and better mental health outcomes.

## 2. Hypothesis Development

According to SDT, intrinsic motivation, extrinsic motivation, and amotivation are the three main motivational orientations. These factors can be arranged along a continuum from high self-determination to low self-determination [19]. In the current study, the amotivation dimension was excluded from further analysis. Amotivation echoes a lack of intention to participate in an activity, featured by a belief of incompetence, lack of value, or disconnection from results [27,32]. Since the main focus of this study is on the association between sports motivation and mental health among Padel players, including amotivation can be less informative, as it highlights a complete lack of motivation rather than the value or type of motivation that can actively contribute to players’ well-being. The study aims to examine how different forms of intrinsic and extrinsic motivation can be related to mental health outcomes such as stress, anxiety, and depressive symptoms. Intrinsic motivation has three main subdimensions (intrinsic motivation to know; intrinsic motivation to accomplish; intrinsic motivation to experience stimulation); similarly, extrinsic motivation has three subdimensions (extrinsic motivation—identified; extrinsic motivation—introjected; extrinsic motivation—external regulation). The sub-domains of intrinsic and extrinsic motivation will be discussed in the following sections.

### 2.1. Intrinsic Motivation to Know and Mental Health

Intrinsic motivation to know describes people with an inherent desire to participate in sport to learn, discover, or promote cognitive development [18]. Within SDT, this type of motivation is recognised as highly autonomous, as it is guided by personal interest and curiosity rather than external rewards or pressures [13,26,32]. When participants are driven to learn and explore their sport, they frequently experience a stronger level of competence and autonomy (two core psychological desires that are steadily recognised as main determinants of emotional well-being and mental health) [6]. The satisfaction of these desires might play a defensive role against mental health disorders, as participants who implement a learning-oriented approach are more likely to have enjoyment, understanding, and individual growth, which might safeguard against mental health symptoms and psychological exhaustion [33]. In this setting, learning-focused involvement can shift attention away from rigid evaluation toward self-improvement, thereby minimising maladaptive rational patterns such as self-blame and failure [34]. Nevertheless, it is important to note that intrinsic motivation to know is not universally related to positive results. Under certain circumstances, it might also be correlated with raised psychological stress. For example, higher intrinsic motivation may be associated with greater investment in skill development and performance, alongside higher self-imposed expectations, frustration when progress is perceived as slow, and greater mental fatigue [20]. In such a setting, the constant pursuit of progress might contribute to stress, anxiety, or even depression, specifically in competitive environments where learning demands are high.

Previous empirical evidence reported these dual associations. While several studies declared that intrinsic motivation is correlated with lower levels of anxiety, depression, and stress [9,35], other research argued that excessive commitment to learning new sports techniques might increase vulnerability to stress and emotional fatigue [36]. Hence, intrinsic motivation to know might operate as a double-edged emotional instrument, subject to the balance between commitment, setting, and support. As a result, we hypothesise as follows:

**H1a.**  *Intrinsic motivation to know is negatively associated with depression symptoms (as a mental health disorder dimension) among Padel players.*

**H1b.**  *Intrinsic motivation to know has a significant correlation with anxiety symptoms (as a mental health disorder dimension) among Padel players.*

**H1c.**  *Intrinsic motivation to know has a significant correlation with stress symptoms (as a mental health disorder dimension) among Padel players.*

### 2.2. Intrinsic Motivation to Accomplish and Mental Health

Intrinsic motivation to achieve describes involvement in sport with the main aim of overcoming one’s own challenges and frequently developing competence [26]. Within SDT, this type of motivation represents a highly autonomous form of self-regulation, in which individuals are driven by the inherent satisfaction associated with personal growth, competence development, and mastery of skills, rather than by external incentives or rewards [13,27]. Such mastery-guided commitment is closely related to self-efficacy [36], conceptualised as one’s belief in one’s ability to successfully implement the actions needed to achieve the desired consequences [37]. High levels of self-efficacy has been regularly acknowledged as a robust emotional resource that can be used to fight against mental health disorders and symptoms of depression, stress, and anxiety by promoting self-confidence, persistence, and adaptive coping methods [10].

Players who are intrinsically motivated to accomplish are likely to adopt mastery-oriented purposes, highlighting learning, progress, and effort rather than seeking social comparison or consequence-based success [38]. Studies grounded in Achievement Goal Theory argue that mastery-oriented players tend to assess challenges as opportunities for growth rather than a threats to self-worth, which might be related to a lower emotional distress and higher emotional stability [39]. This coping mechanism can minimise fear of failure and performance-linked anxiety [40]. Nonetheless, it is important to acknowledge that intrinsic motivation to accomplish might also have adverse relationships with mental health under certain conditions. When the interest in mastery becomes exceptionally intense or severe, players might target perfection, which can intensify self-induced pressure. In such circumstances, the constant striving for perfection might be related to frustration, stress, and emotional stress, specifically when progress is slow or when expectations are not met [41].

Previous empirical evidence confirmed this dual relationship. While intrinsic motivation to accomplish has repeatedly been correlated with higher levels of emotional regulation and lower levels of stress [42,43], other research has argued that intense achievement targeting combined with aims of perfection can contribute to anxiety, stress, and depressive symptoms, particularly in high-demand competitive settings [5,40]. Hence, we hypthesise as follows:

**H2a.**  *Intrinsic motivation to accomplish is positively associated with depression symptoms (as a mental health disorder dimension) among Padel players.*

**H2b.**  *Intrinsic motivation to accomplish is positively associated with anxiety symptoms (as a mental health disorder dimension) among Padel players.*

**H2c.**  *Intrinsic motivation to accomplish is positively associated with stress symptoms (as a mental health disorder dimension) among Padel players.*

### 2.3. Intrinsic Motivation to Experience Stimulation and Mental Health

Intrinsic motivation to experience stimulation describes involvement in sport for the purpose of intrinsic enjoyment, excitement, positive arousal, innovation, and deep emotional experiences [27]. Through the lens of SDT, this motivational factor aligns with a highly autonomous type of engagement, closely related to affective well-being and temporary positive feelings [21,26]. Players who are driven by stimulation satisfy their desire for autonomy and competence while improving emotional strength, which are key elements in the preservation of mental health [10]. From a psychophysiological standpoint, stimulation-oriented participants trigger neurobiological reward mechanisms, including the release of serotonin, endorphins, and dopamine (neurotransmitters that regulate mood), which are correlated with good mood and reduced stress responses [44]. This mechanism can further contribute to immediate pleasure and longer-term psychosocial regulation, allocating intrinsically stimulating physical activity as a defensive element against depressive symptoms and stress [20].

Previous empirical evidence confirmed this argument, reporting that enjoyment and positive outcomes during physical activity are mostly correlated with lower levels of anxiety, depression, and stress [8,45]. Furthermore, stimulation-based involvement can facilitate emotional detachment from daily stressors and may facilitate flow experiences, both of which are correlated with higher levels of well-being and reduced emotional stress [46,47]. Nonetheless, it is critical to acknowledge that intrinsic motivation to experience stimulation might also have potentially adverse relationships under certain environments. Higher levels of stimulation-seeking may be associated with greater commitment to sport participation and a stronger emphasis on excitement relative to psychological resilience. In such circumstances, a lack of stimulation (e.g., during rest periods or during poor performance) might cause irritability, psychological fluctuations, or intensified stress [24]. Hence, we hypothesise as follows:

**H3a.**  *Intrinsic motivation to experience stimulation is negatively associated with depression symptoms (as a mental health disorder dimension) among Padel players.*

**H3b.**  *Intrinsic motivation to experience stimulation is negatively associated with anxiety symptoms (as a mental health disorder dimension) among Padel players.*

**H3c.**  *Intrinsic motivation to experience stimulation is negatively associated with stress symptoms (as a mental health disorder dimension) among Padel players.*

### 2.4. Extrinsic Motivation—Identified Regulation and Mental Health

Identified regulation embodies a comparatively autonomous form of extrinsic motivation in which individuals engage in sport activities because they personally value outcomes commonly associated with participation, such as better health, stronger social relationships, and opportunities for personal development [11]. Within SDT, identified regulation describes a high level of internalisation, in which external targets are integrated into the self and are consistent with personal values [26,32]. Accordingly, behaviour guided by identified regulation is typically experienced as volitional rather than pressured, allowing people to derive emotional benefits similar to those associated with intrinsic motivation. From a mental health standpoint, identified regulation can act as a factor that is associated with lower levels of mental health symptoms by facilitating sustained commitment to sport and higher levels of emotional well-being. When people recognise sport engagement as meaningful and personally significant, they tend to experience a sense of purpose and self-consistency, which reduces the symptoms of depression and emotional distress [12,33]. This internalised form of motivation has been associated with higher self-esteem, more adaptive coping strategies, and lower levels of stress and anxiety [26].

Nevertheless, it is significant to acknowledge that identified regulations might also have adverse consequences under certain environments. When the valued consequences of sports engagement become exceedingly rigid, people might have a feeling of obligation or pressure to sustain engagement, even in the case of fatigue, injury, or highly competing demands. In such settings, the initial autonomous motivation might be changed to a more controlled state, causing internal conflict and emotional stress.

Previous empirical evidence supported this dual relationship. While identified regulation is commonly correlated with superior psychological adjustment, preservation, and emotional resilience, and lower levels of stress and anxiety [9,19,38], studies also argued that intense investment in valued purposes can, under certain conditions, contribute to stress and emotional fatigue, specifically when expectations are unfulfilled or when commitment becomes inflexible [42]. Thus, we hypothesise as follows:

**H4a.**  *Extrinsic motivation—identified regulation has a significant correlation with depression symptoms (as a mental health disorder dimension) among Padel players.*

**H4b.**  *Extrinsic motivation—identified regulation has a significant correlation with anxiety symptoms (as a mental health disorder dimension) among Padel players.*

**H4c.**  *Extrinsic motivation—identified regulation has a significant correlation with stress symptoms (as a mental health disorder dimension) among Padel players.*

### 2.5. Extrinsic Motivation—Introjected Regulation and Mental Health

Introjected regulation is a form of extrinsic motivation in which people engage in sport activities to avoid adverse self-evaluations (e.g., shame or guilt) or to sustain self-worth and ego [13]. From the lens of SDT, introjected regulation partially satisfies main emotional needs, regularly creating internal conflict and tension through sport involvement. From a mental health lens, introjected regulation might have double and context-dependent relationships [10]. On the one hand, a moderate state of introjected motivation might be related to people’s commitment, discipline, and continuity, particularly when intrinsic motives are imperfect [48]. In this vein, the need to avoid shame or to sustain self-worth can reinforce engagement in sport, which might implicitly help minimise stress and enhance mood through systematic physical activity and social participation [19]. On the other hand, introjected regulation is highly correlated with maladaptive emotional consequences [33]. Players guided by such extrinsic motives repeatedly perceive their participation as an obligation rather than a choice, which might be linked with intensified performance anxiety and concern about failure [33].

Previous empirical evidence has confirmed this dual relationship, but it mainly emphasises the adverse consequences. For example, Sheehan [48] argued that controlled types of motivation, involving introjection, are positively correlated with psychological fatigue, stress, and depression symptoms. Likewise, Ref. [34] demonstrated that players who are high in introjected regulation showed elevated levels of anxiety and poorer levels of emotional regulation, specifically under competitive force [36,42]. Significantly, introjected regulation is correlated with conditional self-esteem, in which self-worth depends on performance outcomes and recognised success. Hence, we hypothesise as follows:

**H5a.**  *Introjected regulation is negatively associated with depression symptoms (as a mental health disorder dimension) among Padel players.*

**H5b.**  *Introjected regulation is negatively associated with stress symptoms (as a mental health disorder dimension) among Padel players.*

**H5c.**  *Introjected regulation is negatively associated with anxiety symptoms (as a mental health disorder dimension) among Padel players.*

### 2.6. Extrinsic Motivation—External Regulation and Mental Health

External regulation is framed within SDT as the least autonomous type of motivation, in which players engage in behaviour mainly to gain external rewards, social endorsement, or avoid disapproval, rather than to satisfy personal desires [26,32]. Externally regulated behaviour reflects an externally recognised locus of causality. It frequently undermines autonomy and the satisfaction of fundamental emotional needs for autonomy, capability, and affiliation (elements closely linked to emotional well-being) [27]. From a mental health standpoint, external regulation might exhibit a dual, context-dependent function [33]. On the one hand, external motivations and social beliefs can create and maintain sport involvement, specifically in the absence of intrinsic motivation [20]. In such conditions, external regulation might be associated with behavioural loyalty, structure, and goal-directed practice, which can, in turn, indirectly enhance one’s mood and decrease stress, and might be linked to higher levels of well-being through repeated physical activity and social involvement [41]. For some players, external rewards might also strengthen self-efficacy and offer short-term emotional benefits. On the other hand, a considerable body of literature argues that external regulation is more consistently associated with maladaptive psychological outcomes. In this vein, because people’s behaviour can usually be guided by external rewards rather than personal motivation, people might experience pressure, reduced levels of autonomy, and psychological tension, which can upsurge vulnerability to anxiety, stress, and depressive symptoms [9,12].

Previous research confirmed this dual pathway but primarily emphasised the adverse association over the long term. While external rewards might reinforce short-term involvement, longitudinal studies argued that relying on external rewards is correlated with higher levels of psychological exhaustion, accumulated stress, and reduced emotional health [49]. Consequently, the associations between external regulation and mental health status depend on the balance among external challenges, personal control, and the wider motivational environment in which sport engagement occurs. Hence, we hypothesise as follows:

**H6a.**  *External regulation is positively associated with depression symptoms (as a mental health disorder dimension) among Padel players.*

**H6b.**  *External regulation is positively associated with stress symptoms (as a mental health disorder dimension) among Padel players.*

**H6c.**  *External regulation is positively associated with anxiety symptoms (as a mental health disorder dimension) among Padel players.*

## 3. Research Methods

### 3.1. Measures

In this study, the proposed model includes six independent variables and three dependent variables. The independent variables represent two broad categories of sport motivation derived from Self-Determination Theory. Intrinsic motivation comprises three sub-domains: intrinsic motivation to know, to accomplish, and to experience stimulation. Similarly, extrinsic motivation comprises three sub-domains: identified regulation, introjected regulation, and external regulation. The dependent variables consist of three dimensions of mental health disorders: depression, anxiety, and stress.

The present study focuses on depression, anxiety, and stress as the dependent variables because they represent three of the most commonly examined indicators of psychological distress in mental health and sport psychology research [6]. These dimensions capture complementary aspects of negative psychological functioning and have been widely used to assess mental health outcomes among physically active populations [5].

All measurement items were adopted from previously validated and widely used scales reported in the literature. To ensure relevance to the Padel context and facilitate respondent understanding, minor contextual wording adjustments were made where necessary, while preserving the original conceptual meaning and psychometric properties of the constructs. Therefore, the measures used in this study represent contextually adapted versions of established instruments rather than newly developed scales. The Sport Motivation Scale (SMS), first coined by Pelletier et al. [26] and later developed by Vallerand et al. [20], was employed. The SMS was selected because it is one of the most widely used and empirically validated instruments for assessing sport motivation within the framework of Self-Determination Theory. The scale captures multiple dimensions of intrinsic and extrinsic motivation and has demonstrated satisfactory reliability and validity across various sport and exercise contexts. Its multidimensional structure aligns closely with the objectives of the present study, which seeks to examine the differential relationships between distinct motivational regulations and mental health outcomes among Padel players in Saudi Arabia. The scale, which has six dimensions (each has four items) of motivation, was operationalized following the suggestions of Pelletier et al. [26]; participants were asked to indicate to what extent each of the scale items corresponded to one of the reasons for which they were presently practicing Padel on a 7-point Likert scale, where 1 meant “does not correspond at all” and 7 meant “corresponds exactly”. As per Lovibond and Lovibond [27], a shorter version of the DASS-21 (Depression, Anxiety, and Stress Scale–21 Items) was employed to measure mental health disorders with three factors (each with 7 items). Participants were required to evaluate their level of agreement using a 4-point Likert scale, where 0 revealed “no agreement” and 3 suggested “a high level of agreement”. To ensure that the scale had adequate content validity, it was assessed by 25 experts (10 professors and 15 Padel players). The 25 experts demonstrated that all the scale questions were clear, adequate, and relevant. Accordingly, no corrections were needed [50].

It is worth noting that different Likert-scale formats were used across constructs, as each measurement scale was retained in its originally validated form to preserve its established psychometric properties and ensure consistency with prior studies. Within the PLS-SEM approach, all indicators were standardised during model estimation via an algorithmic procedure that generates comparable latent variable scores regardless of the original response scale [51]. Therefore, differences in Likert scale ranges do not affect parameter estimation or the model’s structural relationships.

As the dependent and independent variables were collected from the same respondents (Padel players), “common method bias” (CMB) might be a concern. To assess the probability of CMB occurrence, “Harman’s single-factor method” (threshold < 50%) was used [52]. The results indicated that a single factor explained 33.21% of the overall variance, below the 50% threshold. Furthermore, all “variance inflation factors” (VIFs) were found to range from 1.218 to 4.084 (VIFs should be <5.0) [53], below the suggested threshold of 5, indicating no multicollinearity concerns. Although multicollinearity diagnostics indicated that all variance inflation factor (VIF) values were within acceptable limits, some values approached the upper threshold, suggesting a degree of shared variance among certain motivational constructs [53]. This is not unexpected, given that Self-Determination Theory conceptualises motivational regulations along a continuum, with adjacent forms of motivation (e.g., identified and introjected regulation) theoretically and empirically related [52].

### 3.2. Data Collection Process

The study data was obtained by employing a cross-sectional quantitative survey approach. This approach is adequate for exploring relationships among several different variables at a single point in time. A non-probability sampling design (convenience and snowball approach) was used due to the lack of a complete sampling frame for Padel players in SA. The survey was developed and distributed via an online link and was distributed through common social media platforms (i.e., WhatsApp, X, and Instagram). Participants were also encouraged to widely share the survey link on their social networks, thereby facilitating easy access and participation.

#### Participants

The target population consisted of Padel players in Saudi Arabia (SA), given the sport’s rapid growth and acceptance in this setting. The data collection process took place over a two-month period (January–February 2026) to ensure satisfactory coverage and response rates. Inclusion criteria were: (a) age 17 years or older, (b) current participation in Padel in Saudi Arabia, and (c) willingness to complete the online survey. Exclusion criteria were: (a) incomplete responses and (b) non-Padel players. As shown in Table 1, a total of 475 valid responses were obtained. Table 1 summarises the demographic characteristics of the sample. The sample was predominantly male (84.8%), young (70.9% aged 20–35 years), and highly educated (89.1% holding a bachelor’s degree or higher). Regarding playing experience, 80% identified as recreational players and 20% as experienced/competitive players. Furthermore, the results show that participants reported a wide range of playing experiences. Specifically, the majority of respondents had 1–5 years of experience (*n* = 356, 75%), followed by those with more than 5 years of experience (*n* = 119, 25%).

The online survey design enabled inexpensive data collection across a wide geographic area while safeguarding anonymity and minimising social desirability bias.

The completion of the designed online questionnaire was entirely voluntary, and respondents were informed of the study’s main purposes and assured of their confidentiality and anonymity. Furthermore, informed consent was obtained before each contribution. The study protocol was reviewed and approved by the Institutional Review Board of King Faisal University (Approval No. KFU-REC-2025-DEC–ETHICS3883, dated 21 December 2025). All procedures followed the ethical standards of the Declaration of Helsinki. The collected sample size was considered adequate for subsequent data analyses, as it met the recommended requirements for Structural Equation Modelling (SEM) [54,55]. For studies conducted at a 95% confidence level with a 5% margin of error, and where the population size is unknown or very large (i.e., exceeding 100,000), a minimum sample size of approximately 384 participants is generally considered sufficient to ensure statistical representativeness and acceptable power. In such cases, researchers typically adopt the next-highest available value in standard sample-size determination tables. In the present study, the achieved sample size of 475 respondents exceeds this recommended threshold, thereby providing stronger statistical power and enhancing the robustness, reliability, and generalizability of the findings.

### 3.3. Data Analysis Techniques

The current study model is complex, as it has six independent reflective latent variables and three dependent reflective latent variables. Likewise, the main purpose of the current study is to investigate the relationships among the structured, interrelated variables rather than to confirm a current theoretical model. Specifically, the model was selected because it aligns with the study’s theoretical framework and research objectives. Hence, the “Partial Least Squares Structural Equation Modeling” (PLS-SEM) method was deemed adequate [56], and thus we used SmartPLS software v4. More specifically, PLS-SEM was employed in this study due to its suitability for prediction-oriented research and its capacity to handle complex multivariate models involving multiple latent constructs [57]. Unlike covariance-based SEM, which is primarily designed for theory confirmation and model fit assessment, PLS-SEM focuses on maximising the explained variance of endogenous variables, making it particularly appropriate for studies that aim to explore and extend theoretical relationships [58,59]. In addition, PLS-SEM is robust to potential deviations from multivariate normality and performs well with complex models and moderately large sample sizes [60,61]. Given these advantages and the exploratory–predictive nature of the present study, which examines the relationships between sport motivation and mental health outcomes, PLS-SEM was deemed the most appropriate analytical approach. The PLS-SEM technique was run in two successive stages. The first one evaluated the measurement model for convergent validity (“Cronbach’s alpha, item loadings, composite reliability, and AVE”), and discriminant validity (“Fornell–Larcker criterion and the HTMT”). The second stage assessed the structural model path coefficients (β), R^2^, Q^2^, and t-values [61].

## 4. Study Results

### 4.1. First Stage: Measurement Model Evaluation

To evaluate the reliability and validity of the measurement outer model and to identify whether the employed scales were aligned with the proposed theoretical factor structure, the measurement model in PLS-SEM was inspected [61]. As shown in Table 2, the factor loadings (FLs) of the measurement variables ranged from 0.713 to 0.997, exceeding the suggested threshold of 0.70, indicating strong reliability and obviating the need to eliminate any measurement items from further analysis [60]. Convergent validity was also considered using the “average variance extracted” (AVE). As shown in Table 2, the AVE scores for all factors exceeded the suggested threshold of 0.50 (ranging from 0.612 to 0.982). Furthermore, composite reliability (CR) values and Cronbach’s alpha were all above the suggested thresholds of 0.7. Notably, the AVE values for intrinsic motivation to know (0.982) and extrinsic motivation—introjected regulation (0.969) were exceptionally high, suggesting potential item redundancy. Given that the Sport Motivation Scale (SMS) is a well-validated instrument, these values likely reflect strong internal consistency rather than measurement problems. Also, one item (IMTA_2) had a factor loading of 0.688, slightly below the conventional 0.70 threshold, but it was retained for theoretical consistency with the original SMS framework. These results confirmed the reliability and convergent validity of the measurement model used.

Discriminant validity was assessed using the “Fornell–Larcker criterion” (Table 3) and the “heterotrait–monotrait ratio” (HTMT) (Table 4). According to the Fornell–Larcker criterion, the square roots of the AVEs for each factor were found to be superior to the correlations between that factor and other factors in the model [61]. Furthermore, all HTMT scores were under the suggested threshold of 0.85 [60]. These findings confirmed that discriminant validity was well established in this study.

### 4.2. Second Phase: Structural Model Evaluation and Hypothesis Assessment

The proposed model has a predictive relevance, as indicated by Q2 scores that were well above zero [60]. Likewise, the “standardized root means square residual” (SRMR) value is 0.035, which indicates adequate goodness of fit (GoF) [61,62]. Furthermore, the “Normed Fit Index” (NFI) value is 0.891, indicating an acceptable fit (slightly below the conventional threshold of 0.90 for good fit) [55], supporting the appropriateness of the model fit [56].

**Figure 1:**
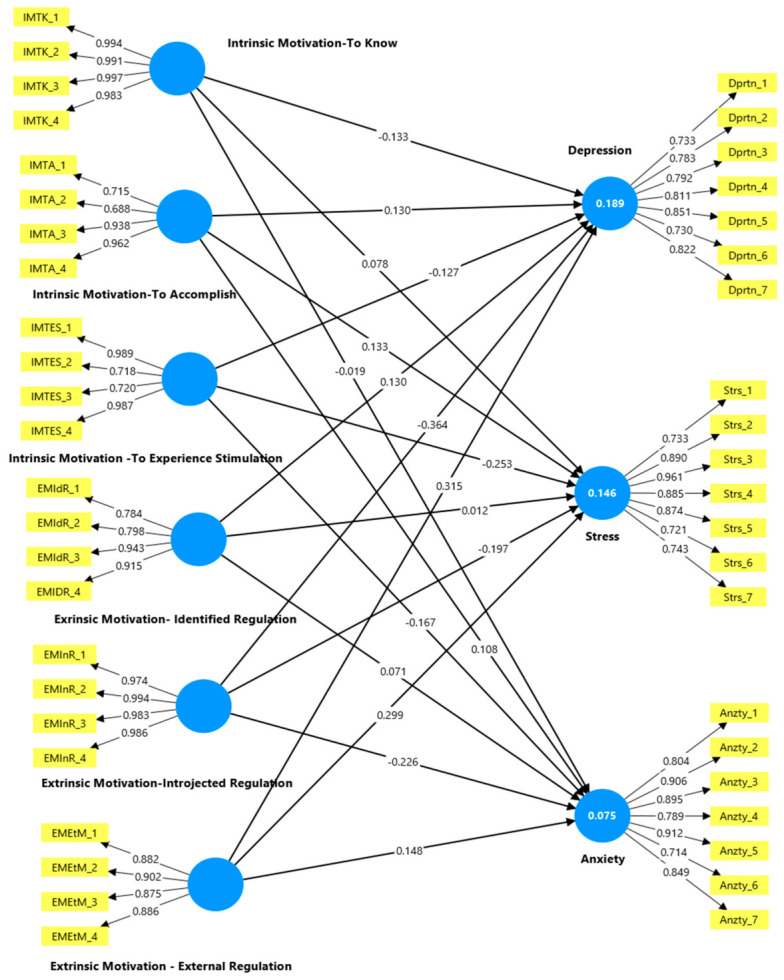
Evaluated model.

After confirming the validity, reliability, and GoF of the tested model and following Henseler et al. [63] ‘s two suggested stages, the research hypotheses were evaluated using t-values, β values, and *p*-values from a bootstrapped resampling method, evaluating the proposed model with 5000 bootstrapped resamples. As seen in Figure 1 and Table 5, intrinsic motivation to know showed a significant association with a lower level of depression (as a mental health symptom) among Padel players in SA (β = −0.133, t = 2.389, *p* < 0.05), supporting H1a. However, intrinsic motivation to know was not significantly associated with stress (β = 0.078, t = 1.894, *p* = 0.058) or anxiety (β = −0.019, t = 0.262, *p* = 0.793), rejecting H1b and H1c. Interestingly, intrinsic motivation to accomplish demonstrated a significant association with an increased level of depression (β = 0.130, t = 1.971, *p* < 0.05), stress (β = 0.133, t = 2.637, *p* <0.05) and anxiety (β = 0.108, t = 1.969, *p* < 0.05), supporting H2a, H2b, and H2c. Moreover, intrinsic motivation to experience showed a significant association with decreased level of depression (β = −0.127, t = 2.426, *p* < 0.05), stress (β = −0.253, t = 2.463, *p* < 0.05), and anxiety (β = −0.167, t = 2.494, *p* < 0.05), supporting H3a, H3b, and H3c.

Concerning the findings of extrinsic motivation factors, the PLS-SEM results reported that extrinsic motivation—identified regulation was not significantly associated with depression (β = 0.130, t = 1.749, *p* = 0.080), stress (β = 0.012, t = 0.184, *p* = 0.854) or anxiety (β = 0.071, t = 0.693, *p* = 0.489), rejecting H4a, H4b, and H4c. However, extrinsic motivation—introjected regulation demonstrated a significant association with decreased levels of depression (β = −0.364, t = 6.739, *p* < 0.001), stress (β = −0.197, t = 3.139, *p* < 0.002), and anxiety (β = −0.226, t = 3.736, *p* < 0.001), supporting H5a, H5b, and H5c. Finally, extrinsic motivation—external regulation demonstrated a significant association with increased levels of depression (β = 0.315, t = 5.898, *p* < 0.001), stress (β = 0.299, t = 7.069, *p* < 0.001), and anxiety (β = 0.148, t = 2.262, *p* < 0.05), supporting H6a, H6b, and H6c.

## 5. Discussion and Implications

The results of the current study revealed several contrasting patterns across the two main dimensions of motivation (intrinsic and extrinsic), providing a nuanced understanding of how different motivational orientations can be associated with mental health consequences among Padel players in SA.

The results offer partial support for the associations between intrinsic/extrinsic motivation and mental health outcomes, providing significant insights within this exploratory research. The PLS-SEM findings reported that intrinsic motivation to know is significantly correlated with decreased (negative) depression symptoms. This suggests that players who participate in Padel out of interest and a desire for learning and skill progression are more likely to show lower depressive symptoms. In the context of Padel in SA (where this sport is rapidly expanding and consistently associated with skill acquisition and social interaction), this result is theoretically aligned with SDT [13]. Learning-oriented participation may reinforce senses of competence, purpose, and autonomy, which are well known to help safeguard against depressive distress.

Additionally, the non-significant association between intrinsic motivation to know and anxiety suggests that motivation to know might not be completely linked with anxiety levels in this setting. Anxiety, specifically in a sports context, is regularly more strongly bound to performance assessment, uncertainty, and social contrast, instead of learning orientation per se [2,5,6]. In athletics, particularly among younger, possibly competitive players, anxiety may be guided more by competitive pressures and situational factors than by intrinsic learning drivers [4,29,40]. This can explain why the intrinsic motivation to know did not translate into significant decreases in anxiety symptoms. Likewise, the association between intrinsic motivation to know and stress symptoms approached significance but remained non-significant (β = 0.078, *p* = 0.058), with a positive correlation that is noteworthy. This pattern might reflect the dual pathway of learning-oriented motivation. While curiosity and skill development can facilitate participation and satisfaction [5], they may also be related to greater cognitive investment, effort, and self-experimentation, particularly in a technically challenging sport such as Padel [23]. From an exploratory perspective, these results suggest that intrinsic motivation to know functions more clearly as a factor associated with lower levels of depressive symptoms than as a controller of anxiety or stress among Padel players in SA. This differentiation emphasises the significance of disaggregating mental health consequences, as different factors (depression, stress, anxiety) might react differently to the same motivational factors.

Additionally, intrinsic motivation to accomplish revealed a significant and positive association with depression, stress, and anxiety. Although intrinsic motivation is usually theorised as helpful, in the present study, intrinsic motivation to accomplish was associated with higher levels of psychological burden. One probable justification is that Padel players who are substantially guided by a continuous achievement and development might advance stronger self-efficacy beliefs and performance predictions. Likewise, in practising sports like Padel, which involves recurrent performance feedback, social comparison, and a competitive interface [24], the target of accomplishment might become linked to ego involvement and fear of deficit. This can cause intensified cognitive and emotional consequences, including reflection, pressure to achieve high performance, and fear of mistakes, which are well-known predecessors of depression, anxiety, and stress [4]. The observed association suggests that intrinsic motivation to accomplish may be linked to a less adaptive psychological profile, whereby striving for mastery is associated with greater emotional strain rather than more favourable mental health outcomes.

In contrast, intrinsic motivation to experience stimulation showed a constant negative correlation with all three mental health symptoms (depression, stress and anxiety). This finding suggests that Padel players who participate for excitement and enjoyment tend to report lower levels of depression, stress, and anxiety. These results are consistent with theoretical expectations, as enjoyment-based involvement is more strongly correlated with affective well-being, stress relief, and emotional regulation [3,4]. In the context of SA, where Padel is regularly practised as a recreational and social activity, stimulation-derived motivation may reinforce positive social bonding and emotional detachment from the daily routine and stress [6]. Additionally, the opposing associations between “to accomplish” and “to experience” emphasise a significant distinction: not all the intrinsic motivation factors are equally beneficial. While all are internally guided, their emotional relationship differs substantially. Motivation to accomplish is goal-guided and effort-oriented, which might be associated with higher levels of pressure, while motivation to experience is affect-driven and enjoyment-oriented, which facilitate relaxation and psychological recovery.

With regard to the extrinsic motivation factor, the findings showed that identified regulation is not significantly correlated with depression, anxiety or stress. Although Padel players might deliberately value the advantages of this sport (i.e., health enhancement, good social relations, or personal advancement), such value-guided engagement alone might be insufficient to have a significant association with mental health consequences. In this setting, identified regulation might operate more as a cognitive process for justifying participation instead of being a strong psychological driver [49]. For young, well-educated people (the majority of the current study sample), many of whom already know the advantages of physical practice, the recognised value of sport engagement might be taken for granted, thereby limiting its explanatory power for improving mental health symptoms. Furthermore, and more curiously, introjected regulation reported a strong and significant adverse association with depression (β = −0.364), anxiety (β = −0.226), and stress (β = −0.197). These results appear to challenge traditional SDT expectations, which usually link introjected motivation to maladaptive consequences [41]. However, in the current study’s specific context, introjected regulation might serve as a functional motivational mechanism. For young adults in SA, feelings of inner obligation, a desire to sustain self-worth, or an avoidance of guilt might encourage steady practice in Padel, thereby facilitating physical practice and social involvement, which are well-known factors in reducing psychological distress. In other words, while introjected regulation is, in theory, “controlled,” it might, in practice, offer a structured motive that helps people sustain social connections and remain continuously active, specifically in a recreational sport like Padel. For a population described as highly educated and having performance-guided lifestyles, such pressures might be normalised and adaptive to some degree, contributing to enhancing mental health consequences. These findings suggest that the association between introjected regulation and the study variables may be context-dependent.

Finally, as predicted, external regulation reported a strong positive correlation with depression symptoms (β = 0.315), stress level (β = 0.299), and anxiety (β = 0.148). This pattern is consistent with SDT, indicating that sport engagement driven primarily by external pressure or rewards tends to co-occur with higher levels of psychological distress [48]. In the context of Padel, external pressures such as social comparison, ongoing competition, or the need to meet peers’ expectations might increase emotional strain, particularly among players who are sensitive to social evaluation. Taking into consideration that the majority of the current study samples are adults, external regulation might increase performance anxiety, fear of peer judgement, and psychological exhaustion, specifically when combined with other life challenges (i.e., academic challenges). This finding is consistent with the view that externally controlled forms of motivation are associated with lower perceived autonomy and higher levels of stress. Overall, these results highlighted that not all types of extrinsic motivation function in the same way. While identified regulations appeared neutral, introjected regulation was associated with lower levels of psychological distress, whereas external regulation was associated with higher levels of mental health symptoms. Significantly, these findings underscore the need to consider contextual factors when understanding the motivational mechanisms underlying mental health outcomes. This contextual understanding might contribute to the broader literature by clarifying that the association between motivation type and mental health outcomes is not linear but rather depends on the form of regulation and the context in which it operates.

The findings of this paper have several implications for practitioners, sport organisations, and mental health specialists interested in Padel and similar recreational sports. First, the findings suggested that different forms of sport motivation play a significant role in modelling players’ mental health consequences. Specifically, more self-determined forms of motivation appear to be associated with more favourable psychological profiles, underscoring the importance of facilitating intrinsic enjoyment, personal growth, and volitional engagement in sport participation. From a practical perspective, coaches and sport administrators can use these insights to design training environments that highlight autonomy-supportive practices, positive feedback, and skill mastery rather than excessive external pressure or outcome-based comparison. Such strategies may help enhance adaptive motivation among players, thereby contributing to higher levels of mental well-being and sustained participation in Padel. Moreover, sport psychology interventions can be tailored to identify athletes who demonstrate less self-determined motivational profiles and provide targeted support aimed at restructuring motivational orientations. This may include goal-setting strategies, cognitive reframing, and psychoeducational programmes focusing on the mental health benefits of regular sport participation. Finally, at a broader policy level, sport governing bodies may consider incorporating components of motivation and psychological well-being into community sport development programmes. Such integration could not only encourage greater participation in Padel but may also be associated with more favourable mental health outcomes among recreational sport participants.

## 6. Limitations and Future Opportunities to Research

While this study offers novel insights into the relationship between sport motivation and mental health among Padel players in Saudi Arabia, its findings should be interpreted in light of several limitations. First, the cross-sectional design precludes causal inferences regarding the observed associations [30]. Future studies should use longitudinal or experimental approaches to better understand the causal directions and dynamics between the study variables. Second, the use of non-probability convenience and snowball sampling via social media platforms (WhatsApp, X, Instagram) means that not all Padel players in Saudi Arabia had an equal chance of participation. The sample is substantially imbalanced by gender (84.8% male) and age (70.9% aged 17–35). While this profile aligns with the known demographics of Padel players in Saudi Arabia, as reported by industry sources, the findings may not generalise to female players, older adults, or players who do not actively use social media.

Furthermore, voluntary response bias is possible, as individuals with strong opinions about Padel or mental health may have been more likely to complete the survey. As a result, certain attitudes, motivations, or mental health experiences may be overrepresented in the sample, potentially affecting the observed relationships among the study variables and limiting the generalizability of the findings to the broader population of Padel players. Third, the data were obtained using a self-administered online questionnaire, which may be associated with common method bias (CMB) and social desirability effects [61]. Although Harman’s single-factor test was used as a statistical procedure to assess potential common method bias, it is acknowledged that this approach alone may not fully eliminate concerns about common method variance. More advanced techniques, such as marker variables or latent factor modelling, were not used in the present study. Therefore, given the reliance on self-reported, cross-sectional data, the possibility of common method bias cannot be completely ruled out, and the findings should be interpreted with appropriate caution. Although several procedural measures were implemented, future research can further strengthen scale validity by adopting multi-method approaches, such as behavioural tracking (e.g., playing frequency), assessment of emotional stress indicators, and peer/coach evaluations. Fourth, although the research employed motivation as a multidimensional construct based on SDT, it did take into account other possibly related contextual or psychological variables. Factors such as personality traits, coping mechanisms, group support, training climate, and rivalry level may be interrelated with the employed motivational factors to form players’ mental health states. Additionally, the use of convenience and snowball sampling may introduce self-selection bias, as individuals who chose to participate may differ systematically from those who did not, which may limit the generalizability of the findings to the wider population of Padel players in Saudi Arabia. Although these sampling strategies are often used in sport science research due to accessibility constraints, the present limitations should be considered when interpreting results, as they may influence observed relationships among study variables and limit external validity.

Future research can expand the tested model by including additional moderating or mediating variables to provide more comprehensive insight into the underlying relationships. Prominently, the results of the current study revealed variance associations between the motivational factors, mainly the contrasting associations of “intrinsic motivation to accomplish versus “intrinsic motivation to experience stimulation”, as well as the unpredicted correlations of introjected regulation. These findings highlight the need for future research to further examine the “dual nature” of motivation, whereby certain forms of motivation may be associated with both greater sport involvement and higher levels of emotional pressure. Exploring non-linear associations might provide a deeper understanding of when motivational factors are helpful versus harmful. Finally, qualitative research designs (e.g., focus groups or interviews) can offer richer, context-specific insights into how Padel players recognise and perceive different motivational factors in relation to their emotional and mental health and well-being.

## 7. Conclusions

This cross-sectional study examined associations between multidimensional sport motivation and mental health outcomes among Padel players in Saudi Arabia. The findings indicate that not all forms of intrinsic motivation are uniformly associated with better mental health: while motivation to experience stimulation was consistently correlated with lower depression, stress, and anxiety, motivation to accomplish showed positive correlations with all three distress indicators. Among extrinsic motivations, external regulation was positively associated with poorer mental health, whereas introjected regulation unexpectedly showed negative associations with psychological distress. The identified regulation was not significantly related to any outcome. These results suggest that promoting enjoyment-based, stimulation-focused engagement may be more beneficial for mental well-being than facilitating achievement-oriented or externally pressured participation. Given the correlational design and the predominantly young, male, recreational sample, findings should be interpreted as associations rather than causal effects. Future longitudinal and qualitative research is needed to clarify the direction and mechanisms of these relationships across diverse Padel populations.

## Figures and Tables

**Table 1:** Demographic characteristics of participants (*n* = 475).

Characteristic	Category	*n*	%
Gender	Male	403	84.8%
	Female	72	15.2%
Age	17 to 19 years	43	9.1%
	20–35 years	337	70.9%
	36–50 years	95	20.0%
Education	Secondary or less	52	10.9%
	Bachelor’s degree	304	64.0%
	Postgraduate degree	119	25.1%
Playing experience	Recreational	380	80.0%
	Experienced/competitive	95	20.0%
Years of playing experience	1 to 5 years	356	75%
	More than 5 years	119	25%

**Table 2:** Measurement model results.

Factors	FL	VIF	α	CR	AVE
Anxiety			0.934	0.944	0.708
Anzty_1	0.804	2.135			
Anzty_2	0.906	3.067			
Anzty_3	0.895	3.455			
Anzty_4	0.789	3.365			
Anzty_5	0.912	3.071			
Anzty_6	0.714	2.110			
Anzty_7	0.849	3.710			
Depression			0.894	0.917	0.612
Dprtn_1	0.773	2.134			
Dprtn_2	0.783	2.715			
Dprtn_3	0.792	2.353			
Dprtn_4	0.811	2.336			
Dprtn_5	0.851	3.203			
Dprtn_6	0.730	2.037			
Dprtn_7	0.822	3.290			
Extrinsic Motivation—External Regulation			0.912	0.936	0.786
EMEtM_1	0.882	4.084			
EMEtM_2	0.902	3.100			
EMEtM_3	0.875	2.312			
EMEtM_4	0.886	4.084			
Extrinsic Motivation—Identified Regulation			0.896	0.920	0.744
EMIdR_1	0.915	3.028			
EMIdR_2	0.784	2.220			
EMIdR_3	0.798	2.470			
EMIDR_4	0.943	3.107			
Extrinsic Motivation—Introjected Regulation			0.989	0.992	0.969
EMInR_1	0.974	3.417			
EMInR_2	0.994	3.014			
EMInR_3	0.983	3.346			
EMInR_4	0.986	1.470			
Intrinsic Motivation to Accomplish			0.869	0.900	0.697
IMTA_1	0.715	2.426			
IMTA_2	0.688	2.422			
IMTA_3	0.938	2.329			
IMTA_4	0.962	2.904			
Intrinsic Motivation to Experience Stimulation			0.921	0.920	0.747
IMTES_1	0.989	1.218			
IMTES_2	0.718	3.960			
IMTES_3	0.720	3.268			
IMTES_4	0.987	1.621			
Intrinsic Motivation to Know			0.994	0.996	0.982
IMTK_1	0.994	3.148			
IMTK_2	0.991	3.571			
IMTK_3	0.997	1.739			
IMTK_4	0.983	1.197			
Stress			0.925	0.941	0.696
Strs_1	0.733	1.911			
Strs_2	0.890	3.516			
Strs_3	0.961	3.855			
Strs_4	0.885	3.421			
Strs_5	0.874	2.934			
Strs_6	0.721	2.313			
Strs_7	0.743	2.076			

Note 1: FL = Factor loadings, α = Cronbach’s alpha, C.R = composite reliability, AVE = average variance extracted, VIF = variance inflation factor. Note 2: IMTA_2’s loading is 0.688; all other loadings exceed 0.70.

**Table 3:** Fornell–Larcker calculations.

	1	2	3	4	5	6	7	8	9
1—Anxiety	0.841								
2—Depression	0.709	0.783							
3—Extrinsic Motivation—Identified Regulation	0.032	0.108	0.863						
4—Extrinsic Motivation—External Regulation	0.103	0.244	0.621	0.887					
5—Extrinsic Motivation—Introjected Regulation	−0.172	−0.231	0.389	0.301	0.984				
6—Intrinsic Motivation to Experience Stimulation	−0.104	−0.057	0.434	0.310	0.293	0.864			
7—Intrinsic Motivation to Accomplish	0.018	0.060	0.363	0.345	0.302	0.548	0.835		
8—Intrinsic Motivation to Know	0.022	0.006	0.513	0.360	0.111	0.373	0.356	0.991	
9—Stress	0.682	0.668	0.100	0.243	−0.127	−0.111	0.070	0.123	0.834

**Table 4:** HTMT calculations.

	1	2	3	4	5	6	7	8	9
1—Anxiety									
2—Depression	0.778								
3—Extrinsic Motivation—Identified Regulation	0.091	0.141							
4—Extrinsic Motivation—External Regulation	0.114	0.251	0.719						
5—Extrinsic Motivation—Introjected Regulation	0.146	0.230	0.423	0.332					
6—Intrinsic Motivation to Experience Stimulation	0.091	0.142	0.533	0.354	0.328				
7—Intrinsic Motivation to Accomplish	0.083	0.100	0.476	0.392	0.312	0.728			
8—Intrinsic Motivation to Know	0.114	0.153	0.579	0.388	0.112	0.434	0.444		
9—Stress	0.725	0.739	0.101	0.249	0.132	0.132	0.095	0.130	

**Table 5:** Hypothesis evaluation.

	β	*t*	*p*	Results
H1a—Intrinsic Motivation to Know -> Depression	−0.133	2.389	0.017	supported
H1b—Intrinsic Motivation to Know -> Stress	0.078	1.894	0.058	not supported
H1c—Intrinsic Motivation to Know -> Anxiety	−0.019	0.262	0.793	not supported
H2a—Intrinsic Motivation to Accomplish -> Depression	0.130	1.971	0.047	supported
H2b—Intrinsic Motivation to Accomplish -> Stress	0.133	2.637	0.032	supported
H2c—Intrinsic Motivation to Accomplish -> Anxiety	0.108	1.969	0.048	supported
H3a—Intrinsic Motivation to Experience Stimulation -> Depression	−0.127	2.426	0.041	supported
H3b—Intrinsic Motivation to Experience Stimulation -> Stress	−0.253	2.463	0.014	supported
H3c—Intrinsic Motivation to Experience Stimulation -> Anxiety	−0.167	2.494	0.044	supported
H4a—Extrinsic Motivation—Identified Regulation -> Depression	0.130	1.749	0.080	not supported
H4b—Extrinsic Motivation—Identified Regulation -> Stress	0.012	0.184	0.854	not supported
H4c—Extrinsic Motivation—Identified Regulation -> Anxiety	0.071	0.693	0.489	not supported
H5a—Extrinsic Motivation—Introjected Regulation -> Depression	−0.364	6.739	0.000	supported
H5b—Extrinsic Motivation—Introjected Regulation -> Stress	−0.197	3.139	0.002	supported
H5c—Extrinsic Motivation—Introjected Regulation -> Anxiety	−0.226	3.736	0.000	supported
H6a—Extrinsic Motivation—External Regulation -> Depression	0.315	5.898	0.000	supported
H6b—Extrinsic Motivation—External Regulation -> Stress	0.299	7.069	0.000	supported
H6c—Extrinsic Motivation—External Regulation -> Anxiety	0.148	2.262	0.024	supported

Note: β = path coefficients; *t*: T-value; *p*: significance level.

## Data Availability

The original contributions presented in the study are included in the article; further inquiries can be directed at the corresponding author.
